# Simulation of Polymer Fractal Formation Using a Triangular Network Growth Model

**DOI:** 10.1021/acs.langmuir.4c02939

**Published:** 2024-09-27

**Authors:** Kenneth Mulder, Hannah Heierhoff, Sophia M. Lee, Jeannie Ji-Ying Tsou, Wei Chen

**Affiliations:** † Department of Mathematics and Statistics, Mount Holyoke College, South Hadley, Massachusetts 01075, United States; ‡ Department of Chemistry, Mount Holyoke College, South Hadley, Massachusetts 01075, United States; § School of Natural Sciences, Hampshire College, Amherst, Massachusetts 01075, United States

## Abstract

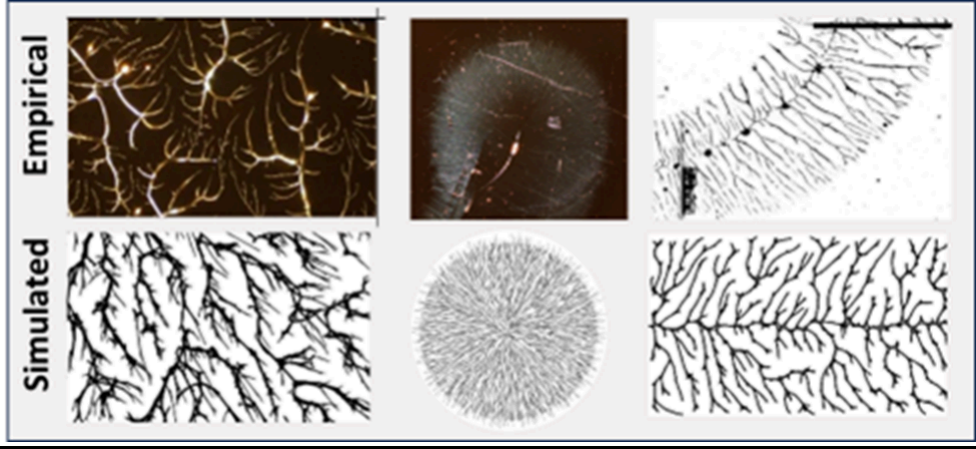

Fractal formation in spin-coated thin-film polymers is of experimental and theoretical interest. Modeling the determinants and dynamics of this process will deepen our understanding of polymer aggregation and the predictability of thin-film structures. This is especially true if the model used has readily interpretable parameters and has been demonstrated to yield a close match to experimental processes under a variety of conditions. In this work, we adapted and applied a relatively new model of fractal growth comprised of a spreading and contracting triangular network, to model spin-coated, thin-film polymers made of poly(vinyl alcohol) on polydimethylsiloxane substrates. We drew clear connections between model parameters and the process of polymer aggregation and we demonstrated the ability of the model to simulate fractal formation under a wide variety of conditions including varying the degree of hydrolysis of the polymer, changing the spin-coating process, and solvent annealing and reforming of polymer fractals under different drying conditions. We also showed how the model is able to replicate idiosyncratic experimental settings yielding novel fractal patterns.

## Introduction

Thin films produced by the spin coating of various polymer solvents demonstrate a diversity of morphological and physiological properties.^1,2^ Under a variety of empirical conditions, polymers aggregate into diffuse structures with a fractal dimension.^3−6^ Wang et al.^7^ surveyed several recent reports of fractal formation in Langmuir–Blodgett thin films and in Langmuir monolayers. The specific processes underlying such aggregation are complex and not well understood, but it is important to materials science that we learn how to manipulate the fractal structure of materials and understand the relationship between fractal characteristics and other materials properties.^7^

The fractal formation process has been modeled using a variety of mathematical structures,^8^ most notably the Diffusion Limited Aggregation (DLA) model^9,10^ as well as Cluster–Cluster Aggregation (or Cluster DLA)^11,12^ and L-system dialectics.^13^ Amir et al.^13^ note that to obtain fractals in a laboratory setting, particles exhibiting Brownian motion are a necessity which is precisely the underpinnings of the DLA model. Spin-coated, thin-film polymers made of poly(vinyl alcohol) (PVOH) on polydimethylsiloxane (PDMS) substrates and reported in Qi et al.^3^ provide a particularly clear example of 2D fractal morphologies that are amenable to assessment using models like the DLA model. Rapid drying by spin-coating leads to polymer bonding in a kinetically trapped state and fractal dimensions have been observed ranging from ∼1.5 to ∼1.8^3^.

In this work, we demonstrate the ability of a new type of fractal growth model based on an expanding triangular network to simulate polymer nucleation and crystallization under a variety of experimental conditions. In particular, we use our model to simulate two types of PVOH thin films (88% and 99% hydrolyzed) spin-coated on silicon-wafer-supported PDMS substrates and produced under several different experimental regimes. The hydrophobicity and mobility of the high molecular weight PDMS substrates cause the PVOH films to dewet and form micron-sized fractal features.^5^ For the purpose of developing our simulation model, we shall abstract the following key elements of the polymer aggregation and bonding process:

1). Polymer strands begin in a relatively homogeneous 2D mass prior to spin coating.
2). When dewetting via spin coating commences, strands coalesce around randomly and uniformly distributed nucleation sites to form initial aggregates.
3). The initial aggregates attach and form bonds as the polymer is further dewetted.
4). Once bonds have formed but are not yet fully dry, they exert an attractive force, causing the mass to contract.
5). Once enough moisture has been removed, the mass becomes stationary.

In 2024, Mulder et al.^14^ introduced a model based on a simultaneously expanding and contracting triangular network that has many similarities to the process described above. As part of their exposition of this new model, they explored similarities between the model process and resulting fractal output and the production and observed morphologies of spin-coated thin-film polymers from aqueous solution.^14^ Model parameters corresponded with identifiable elements of the polymer growth process and the resulting structures were similar in fractal dimension and lacunarity. However, this exploration was only preliminary. In this paper, we seek to fully develop this model as a simulation model for a specific type of polymer fractal development. We seek to demonstrate not only how the model is able to simulate the standard spin-coating process for PVOH thin films, but we also hope to demonstrate the ability of the model to replicate various modifications of the production process through reasonable adjustments of model parameters. If the model is able to simulate this fractal growth process under a variety of conditions, including some idiosyncratic experimental contexts, then there is reason to believe the model will abet scientists in proposing and testing hypotheses about the underlying processes of polymer bonding.

## Experimental Section

First, we provide an overview of the triangular growth model. More details about the model, including different descriptive statistics and how they vary with model parameters, can be found in the original paper.^14^ Simulations were conducted using the software NetLogo.^15^

The model begins with 10^5^ particles uniformly randomly distributed on a disk with radius set as to provide a predetermined average particle density (). Particles represent nucleation sites at a prebonding level of aggregation. Such an assumption is necessitated by the scale at which the model operates in which each particle must represent a large number of polymer molecules. Additionally, in accordance with crystal nucleation and growth theory, these nuclei represent the critical size aggregates must achieve above which growth becomes irreversible.^16^ The critical size is affected by the interfacial energy, or the energy difference between a surface and a bulk solute molecule.

The three particles closest to the center are joined to form an initial triangle. Triangles represent the bonds between nuclei. Each triangle edge is originally set to be active meaning it has the capacity to form a new bond (triangle) with a previously unattached particle.

Each time step, every active edge has a given probability (*bonding-rate*) to attempt to form a new triangle by connecting to an unattached particle. If it is chosen for bonding, it finds the nearest unattached particle (distance equal to the average distance to the segment’s end points), and if that distance is below a given threshold (*bonding-range*), it forms a new triangle by connecting both of its end points to that particle. Regardless of whether or not a new bond is formed, the edge is deactivated and will not be able to bond in the future.

**Figure 1:**
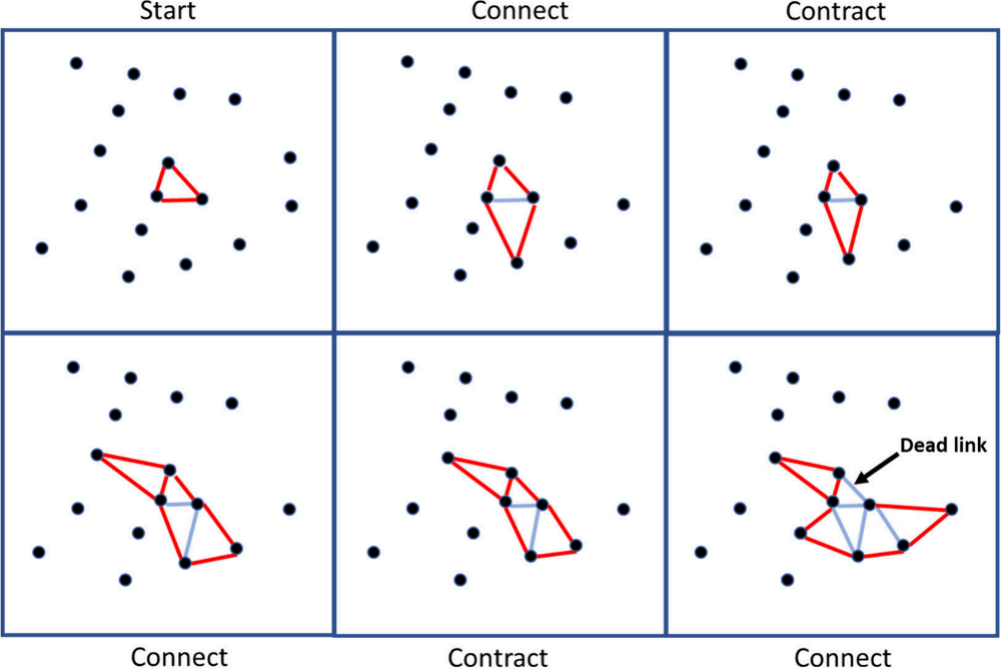
Stylized representation of the triangular growth model modified from Mulder et al.^14^ Alternate frames show the growth and contraction steps. During the growth step, selected edges have the chance to bond with the nearest particle assuming it is close enough. The edge is then deactivated and cannot seek to bond again. Between connecting steps, the network experiences contraction in the direction of the links. Reproduced with permission from ref (14) under the Creative Commons Attribution 4.0 International (CC BY) license, copyright 2024 Mulder et al.

Once this growth step is complete and all selected edges have attempted to bond, some particles will experience a contraction step which causes their position to change. Particles become mobile when they are first connected to the network and remain mobile for a set period of time (*mobility*). All mobile particles experience a pull toward each of their neighbors proportional to the distance between them (as if they were connected by springs). If each of the edges connecting a point to its neighbors is treated as a velocity vector, then each particle experiences a displacement equal to 0.005 times its total velocity. Figure 1 demonstrates the alternating growth and contraction phases.

The model has four parameters that have natural connections to the polymer aggregation processes—*bond-range, bond-rate, density*, and *mobility*. These parameters affect model dynamics and the final morphology of the network. *Bond-range* determines the maximum distance at which particles are able to attach to the growing mass and is given in units where one unit is equal to 2.1 *μm*. *Bond-rate* is the proportion of active edges that seek to form new bonds and controls how quickly bonds seek to form and affects the variability in the structure since, at low levels, more contraction occurs between bonding which generally results in more dead edges. A *bond-rate* of 0.03 means 3% of active bonds seek to form new bonds each tick. Both *bond-range* and *bond-rate* are assumed to be constant across all empirical settings in this paper.

*Mobility* reports the number of timesteps after bonding that a particle experiences contraction and controls the amount of contraction the mass undergoes and is assumed to be proportional to the amount of time a polymer structure takes to dry. We will describe multiple experimental variations that can impact the drying time and thus necessitate a change in this parameter. *Density* (in particles per square unit) controls the density of the initial distribution of particles (nuclei) in the plane. We believe this is affected by two different empirical modifications which will be described later. To conserve overall mass, when *density* is increased, we decrease the width of the triangle edges proportionally.

While we were able to translate the spatial scale in the model to the observed physical scale, this is not the case for the temporal scale. We do not know experimentally the time frame over which bonding occurs, though we do know it is very short (∼ one second or less). Thus, we cannot report the amount of time represented by one tick.

Figure S1 shows one example of network growth at six time periods including the final structure. Figure S2 shows how the final model structure changes in response to changes in density and mobility. Since the initial bond is located in the center, the model exhibits radial growth. This simulates the fact that spin-coating, by affecting when and in what direction particles are able to bond, results in a radial fractal pattern. This is a consequence of the dewetting process, and we describe variations on this later. Prior to dewetting, nuclei do not bond with each other because they are further apart, surrounded by solvent molecules, and diffuse more slowly than individual molecules.

We use our model to simulate a wide variety of experimental contexts, all of which are variants of the following process. We dispensed 400 μL of 0.1 wt % PVOH^99%H^ aqueous solution onto a PDMS^49k^ (MW = 49 kDa) substrate secured on the spin coater stage. After 1 min, the sample was spun at 6000 rpm for 1 min under nitrogen. The sample was stored in a desiccator (CaSO_4_) overnight prior to characterization. We refer to this as our base case. Micrographs of the fractal morphology were produced at 500× magnification unless otherwise specified. To simulate this base case, model parameters were set as follows: *bond-range* = 2.2, *bond-rate* = 0.03, *mobility* = 200, and *density* = 1.0. To simulate variations in the experimental context, we varied *mobility* and *density* as described below. *Bond-range* and *bond-rate* remained constant throughout our simulations.

We explored several variants of this experimental context and corresponding simulations. The experimental setup for each of these variants is described in the results section so as to collocate the experimental design with the relevant model details and output. Model parameters are reported for each context and we also report any necessary changes made to model structure to simulate additional processes.

Primary model validation depended on observed characteristics of the network structure of the images including the density of branching and the distribution of branch lengths. At this time, we do not have a method for measuring these features quantitatively from the images, but we can observe changes in these features between different experimental settings and different parametrizations of the model. If these observed changes are clear and consistent between experimental and simulated output, this is evidence for a correlation between underlying processes.

Two image analysis statistics were also used to compare simulation output to micrographs of polymers. We measured and compared the Minkowski-Bouligan (or box-counting) dimension^17^ of corresponding binarized images using the Fraclac plugin^18^ in ImageJ.^19^ The Fraclac plugin uses the box-counting method^20^ to determine fractal dimension by comparing the log-transformed growth in the number of boxes required to cover an image to the decreasing side-length of the box. Since this statistic is affected by the magnification of an image, we maintained a consistent scaling when simulating polymer development and applied the algorithm to images that had been magnified and cropped similarly. We also used ImageJ to measure the percent coverage of corresponding (binarized) images.

## Results

**Figure 2:**
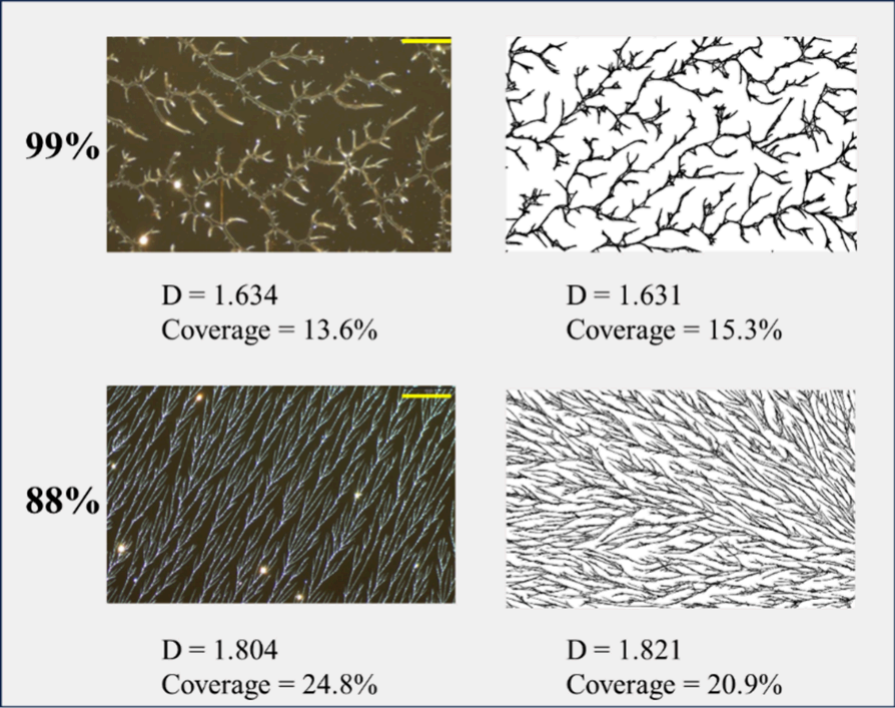
PVOH^99%H^ spin-coated polymers (left) at 500× magnification and model simulations (right). The top row shows results for 99%H and the bottom row shows the results for 88%H. Box-counting fractal dimension and percent coverage are reported for each image. The yellow scale bars have lengths of 20 μm.

Figure 2 (top row) shows one micrograph of a polymer produced under the base-case conditions described above (PVOH^99%^ spun at 6000 rpm) at 500× magnification as well as a simulated polymer produced by the model. Comparison of fractal dimension and percent coverage between the figures as well as a visual comparison of the network structures show a strong structural similarity between the two. Average dimension of the polymer samples was 1.643 with a standard deviation of 0.028 (measured for 14 micrographs taken from 4 samples). Average dimension of the simulated images was 1.647 with a standard deviation of 0.0073 based on a sample of 10 images.

The first variant we explored is experimentally the same as for the base case except with a PVOH^88%H^ solution. While aggregation in PVOH^99%H^ thin films is primarily driven by hydrogen bonding, aggregation of PVOH^88%H^ is dominated by the hydrophobic effect which has less conformational restrictions.^21^ Initially it was our hypothesis that the primary difference between 99% and 88% would be an increase in the bonding rate reflecting the reduced bonding restrictions for 88% versus 99%, and indeed we can achieve plausible simulations of the 88% case by increasing *bond-rate*. However, we have good reason to believe that this is not the correct approach. First, to achieve a good match with the empirical figures, *bond-rate* was increased from 0.03 to 0.5, a 17x increase, which seems extreme. Second, in later experiments in which polymers are annealed in a solvent, we noted that regrowth postannealing of PVOH^99%H^ was very similar to the observed morphologies seen in PVOH^88%H^. However, we were unable to achieve the observed patterns by changing *bond-rate* (more on this below). Changing *density*, however, yielded an effective simulation for both cases.

Thus, the change from 99%H to 88%H is modeled by increasing the density of nucleation sites prior to large-scale aggregation. This is in line with what we would expect from crystallization theory.^16^ The critical size at which nuclei begin to bond is affected by the interfacial energy, or the energy difference between a surface and a bulk solute molecule. Given that 99%H has a much lower bulk energy due to stronger hydrogen bonding, it would have a higher interfacial energy requiring nuclei to be larger prior to crystallization. It is thus conceivable that 88%H has a significantly smaller critical nucleus size and a higher nuclei density than 99%H. For 88%H, we set *density* = 4.0 versus 1.0 for 99%H. The outcome for this is similar to increasing the *bond-rate*. The experimental and simulation results are shown in the second row of Figure 2 where again we see a good correspondence between dimension, density, and network structure. Average dimension of the polymer samples was 1.785 with a standard deviation of 0.017 (measured for 12 micrographs taken from 4 samples). Average dimension of the simulated images was 1.816 with a standard deviation of 0.0039 based on a sample of 10 images.

For our second variant, experiments were conducted using varying volumes of PVOH^99%H^, all much less than the standard 400 μL used in the base case. Quantities ranging from 5–50 μL PVOH solution were spin-coated on PDMS^49k^ at 6000 rpm. Micrographs were produced using a 12.5× magnification.

Two differences were noted using differing volumes. First, the radius of the polymer decreased with volume, but not in a strictly proportional manner. (It should be noted that an unknown quantity of polymer is spun off as evidenced by the polymer formation in the outflow path). Second, due to the decreased radius of the smaller drop sizes, the outer boundary of the drop experienced less centrifugal force when the volume was reduced. As a consequence, smaller volumes took longer to dewet, giving the mass more time to contract.

To simulate these two differences, we first matched the radius by setting the number of particles (at a density of 1.0) to yield the observed radius. Second, we increased *mobility* to achieve a good match in pattern and fractal dimension for the 5 μL trial resulting in a *mobility* of 650. For all other trials, the value of *mobility* was determined as follows. Since centrifugal force is proportional to the radius, and volume is proportional to the radius cubed, we hypothesize that mobility should be inversely proportional to the cube root of the volume of the drop. Using this relationship, for volumes of 10 μL, 20 μL, 30 μL, 40 and 50 μL, *mobility* was set to 516, 409, 358, 325, and 302 respectively.

**Figure 3:**
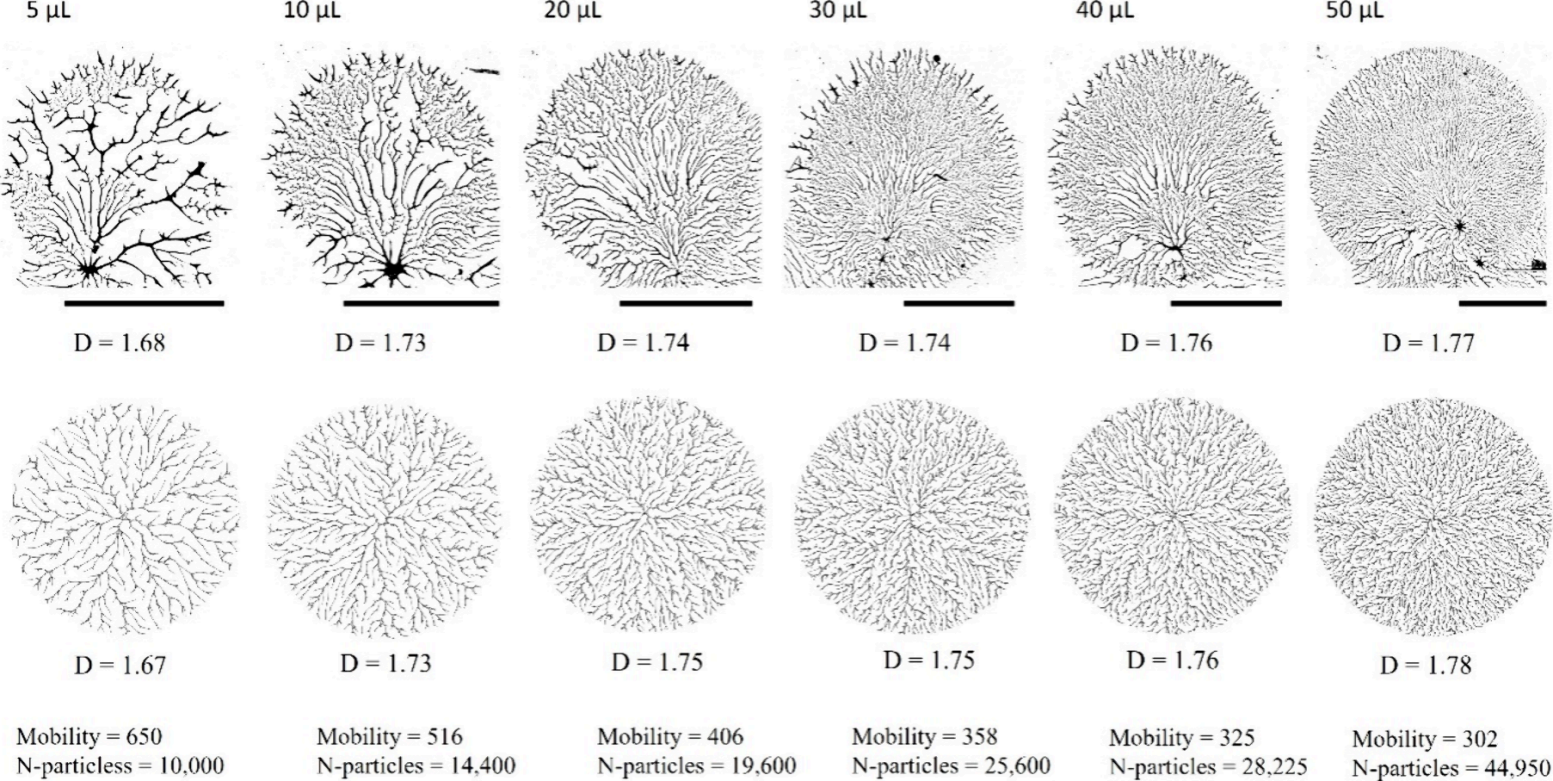
Micrographs of spin-coated polymers produced by applying differing quantities of PVOH^99%H^ (top row) and the corresponding simulation images. Volume of solution applied is shown above each image. Scale bars are 2 mm in length (12.5× magnification). The quantity of PVOH solution applied is shown at the top. Box-counting dimension (D) is shown below each image, and the model parameters used for each trial are shown below. The top row images are reproduced with permission from ref (3), copyright 2019 American Chemical Society.

Qi et al.^3^ reported the results of varying the volume of the polymer solution applied to the substrate prior to spin coating (Figure 3, top row). There is a very clear gradient beyond just a change in radius. In particular, the larger the drop, the finer and denser the structure and the greater the box-counting dimension. The simulations produced by the changes described above are shown in the bottom row of Figure 3 where it can be seen that not only do we match the observed change in dimension but also the observed trend in network density and structure. Standard deviation for the dimensions of the simulated images was 0.00127 estimated from ten simulations per drop size. We were unable to calculate the standard dimension for the empirical images. (Note that the fractal dimension is not consistent with the dimension reported in Figure 2 because of different magnification for the micrographs (12.5x versus 500x) and different drop sizes which affects the box-counting estimate of dimension.)

**Figure 4:**
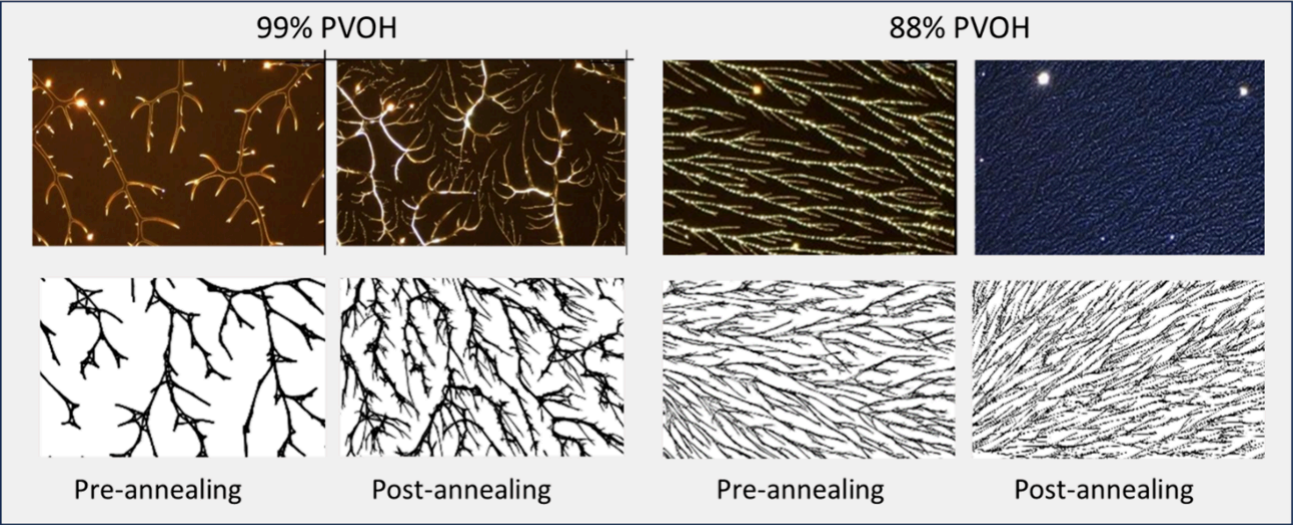
Top Row: Optical images (500× magnification) of PVOH (99%H and 88%H) spin-coated at 6000 rpm (1st and 3rd) and postannealing (2nd and 4th). Bottom Row: Corresponding model simulations created with 10^5^ particles and scaled to match. The only change in model parameters postannealing was to increase the density of aggregated nucleation sites from 1.0 to 4.0 (99%H) or 4.0 to 6.0 (88%H).

Our third experimental variant involved solvent annealing of thin films. Solvent annealing trials using both 99%H and 88%H thin films were conducted by applying a drop of solvent onto a previously spin-coated polymer and then spinning again after 1 min. 400 μL of Milli-Q water was deposited on a PVOH^88%H^-PDMS^49k^ or PVOH^99%H^-PDMS^49k^ sample (prepared using adsorptive spin coating at 6000 rpm). After 1 min annealing time, the sample was spun at 6000 rpm for 1 min under nitrogen. Observations of polymers before and after annealing showed that part or all of the polymer dissolved during the 1 min application and that a new fractal structure grew during the second spinning (Figure 4, top row).

We simulate solvent annealing in two steps. First, a set proportion of bonds are removed to simulate the dissolved part of the structure. The proportion was based on observed before-and-after images which clearly show the loss of previous growth as well as new growth. We also used images from the extended drying time context below which show what proportion of the previous structure remains attached across the annealing boundary. Based on these observations, 20% of bonds for the 99%H case are removed and 100% of bonds for the 88%H case (as imaging showed none of the previous structure remaining.)

In the second stage, the dissolved bonds are assumed to produce a new adsorbed layer of polymer. Observations of the new growth in the 99%H case (Figure S3) show that the new nuclei behave very similarly to the 88%H solution, and a similar change postannealing is seen in the 88%H case (new growth being finer and denser). The observed differences between 88%H and 99%H can be achieved in two ways—by increasing the bonding rate^14^ or by increasing the density—but only increasing the density was effective in simulating the secondary growth observed in the annealing experiments, increasing the *bond-rate* was not. Increasing *density* accounted for the secondary growth being both finer (smaller bond width) and denser. This suggests that the liberated polymer had a much higher bulk energy likely due to weaker hydrogen bonding. Thus, it would have a lower interfacial energy requiring nuclei to be smaller prior to crystallization. Therefore, after removing some of the bonds, new particles were added at a *density* of 4.0 for 99%H and 6.0 for 88%H. At this point, the model was allowed to regrow. All external bonds in the remaining structure were set to active (enabling them to bond), but the model was constrained to grow from the inside out due to the spin-coating effect.

Figure 4 shows spin-coated PVOH polymers (99%H and 88%H) at 500× magnification before and after solvent annealing. The bottom rows show corresponding simulation images. The increased density in nucleation sites postannealing seems to capture well the observed empirical changes.

For our fourth experimental variant, we modified the previous context to explore the boundary between the annealed portion of the polymer and the unannealed portion as well as the dynamics of the annealing edge as the solvent retreated through evaporation. Thirty μL of Milli-Q water was deposited on a PVOH^88%H^-PDMS^49k^ or PVOH^99%H^-PDMS^49k^ sample (previously prepared using adsorptive spin coating at 6000 rpm). The dispensed volume did not cover the entire sample as was the case in the prior experiments. In the first trial, after 1 min the sample was spun at 6000 rpm for 1 min under nitrogen. In the second trial, it was spun after 30 min. In the final trial, the dispensed solvent was allowed to fully evaporate by air drying. Micrographs of the annealed samples were produced at 50x, 100x and 500x at both the initial solvent boundary and in the center.

**Figure 5:**
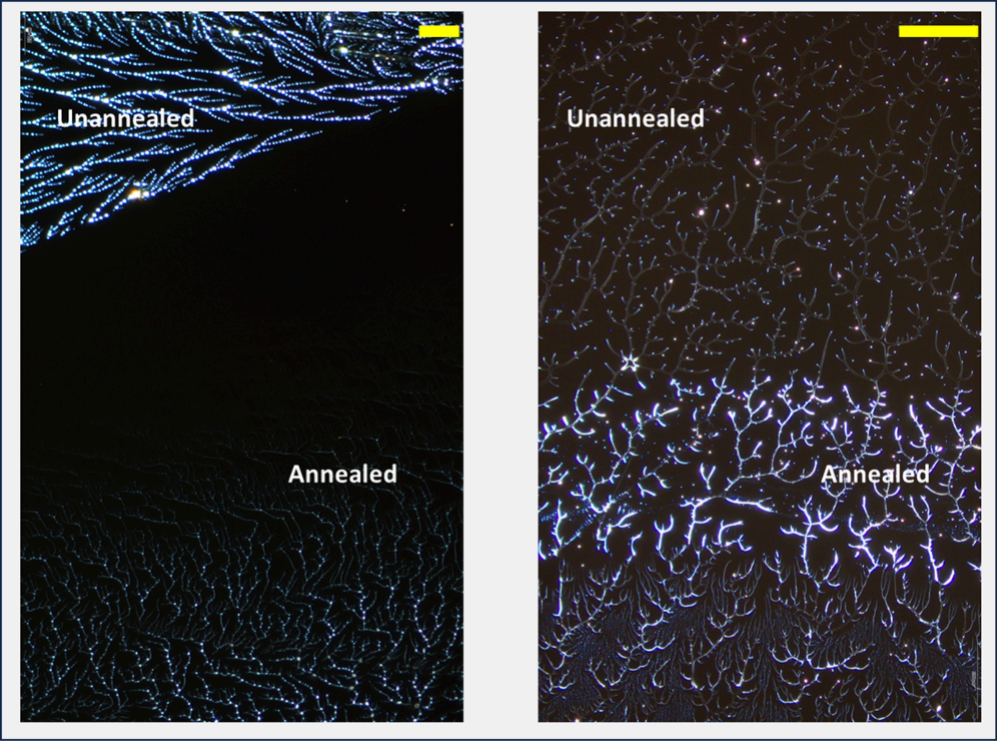
Fractal development and morphology at the drying edge of the applied solvent. One minute after solvent was applied, the film was spun again at 6000 rpm. The micrographs on the left (88%H) were made with 500× magnification and the one on the right (99%H) with 100x. The left and right scale bars have respective lengths of 20 and 200 μm.

Figure 5 shows the drying edge after 1 min. For 88%H, the entire structure appears to have receded with the edge of the drop of solvent as it dries. For 99%H, we see some retraction of the structure, but much of the structure remains. Denser, finer growth in the bottom portion shows annealed regrowth that is also retracting.

**Figure 6:**
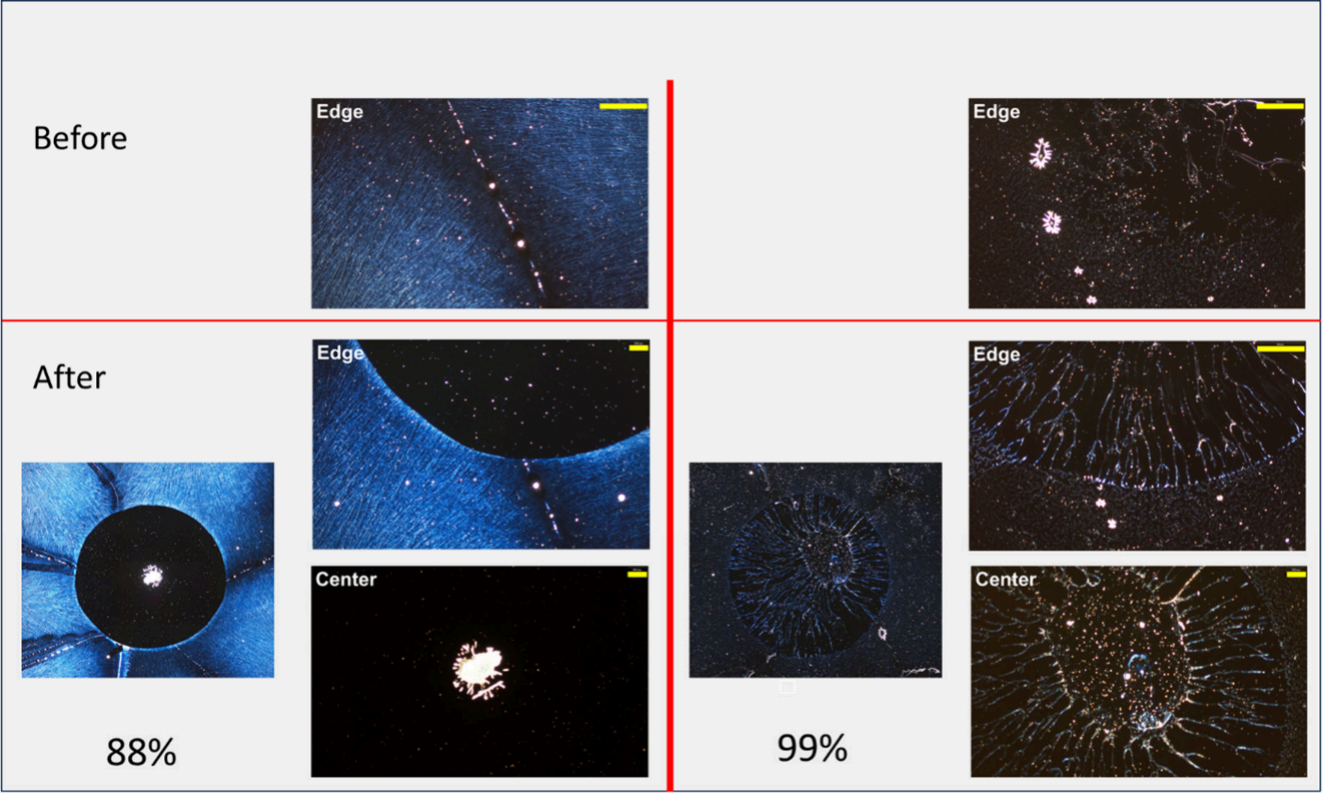
Experimental results from applying a drop of solvent to a polymer thin film and allowing it to air-dry over a period of approximately 2 h. Images on the left were made from an 88%H solution and images on the right from 99%H. The top micrographs show the thin films prior to annealing at 50× magnification. The lefthand images in the lower section are the resulting thin films at 10× magnification. 50× magnification micrographs to the right show the center as well as the boundary of the dispensed solvent after drying. The longer scale bars have lengths of 500 μm while the shorter have lengths of 200 μm.

Figure 6 presents the result of allowing the solvent to evaporate completely without spinning. It shows 88%H and 99%H polymers before and after extended-drying solvent annealing. It took approximately 2 h for the solvent to evaporate. For 88%H, where dissolution of existing bonds is nearly complete, the disk where the solvent was applied is largely devoid of polymer with the exception of the middle. It appears that as the solvent dries and the polymer contracts with it, it reaches a point where polymer density is too high for fractal development. For the 99%H trials, a significant portion of the original polymer remains, forming a series of connections between an inner mass and the outer ring. Extended contraction has created a very sparse structure between the boundary and the inner mass, and once again it appears that the polymer density is too high in the middle for fractal development.

To simulate this process, the portion of the original fractal covered by the dispensed volume (an inner disk smaller than the original) was annealed as described above. Then, to simulate the extended drying along the edge, a drying front was established that moved radially inward from the boundary. Within a given distance of the moving drying front (10 units = 5.5% of radius), particles were considered mobile and experienced contraction. The speed of the front was set so as to take 3 × 10^5^ timesteps to completely retract. This process was stopped at appropriate times to simulate the different drying times with 3 × 10^5^ timesteps equivalent to 2 h.

**Figure 7:**
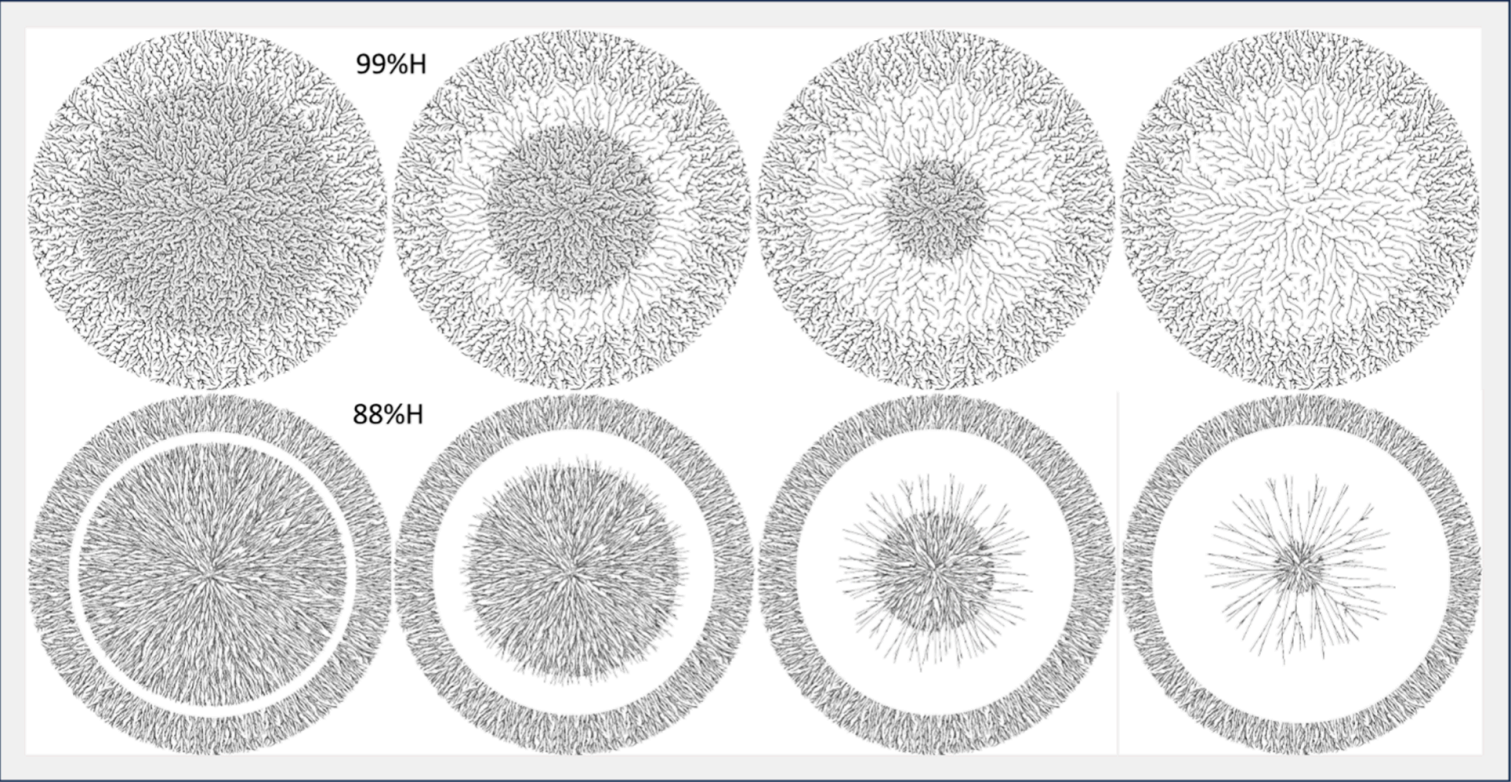
Simulations of polymer annealing assuming a drop of solvent is placed in the middle of a previously created thin film and then dried after differing amounts of time. The top row shows the 99%H simulation and the bottom row the 88%H simulation. Time before drying increases left to right though it is not the same for the top and the bottom. Model modifications and parameters are described in the text.

Figure 7 shows our simulation results for 99%H (top) and 88%H (bottom). Moving from left to right, we see the resulting structures after increasing time lengths simulating what would be seen when spinning the annealed polymer after different amounts of drying up to the point of full air drying. We see similar patterns forming as with the experimental samples due to successive contraction at the drying front. In the 99%H case, sufficient polymer is resistant to desorption as to leave a skeletal structure connecting the inner mass to the original structure. We do not, however, see a mass that persists in the center. In the 88%H, we do see a growing ring that is devoid of particles, and there are recalcitrant structures in the center of the mass.

**Figure 8:**
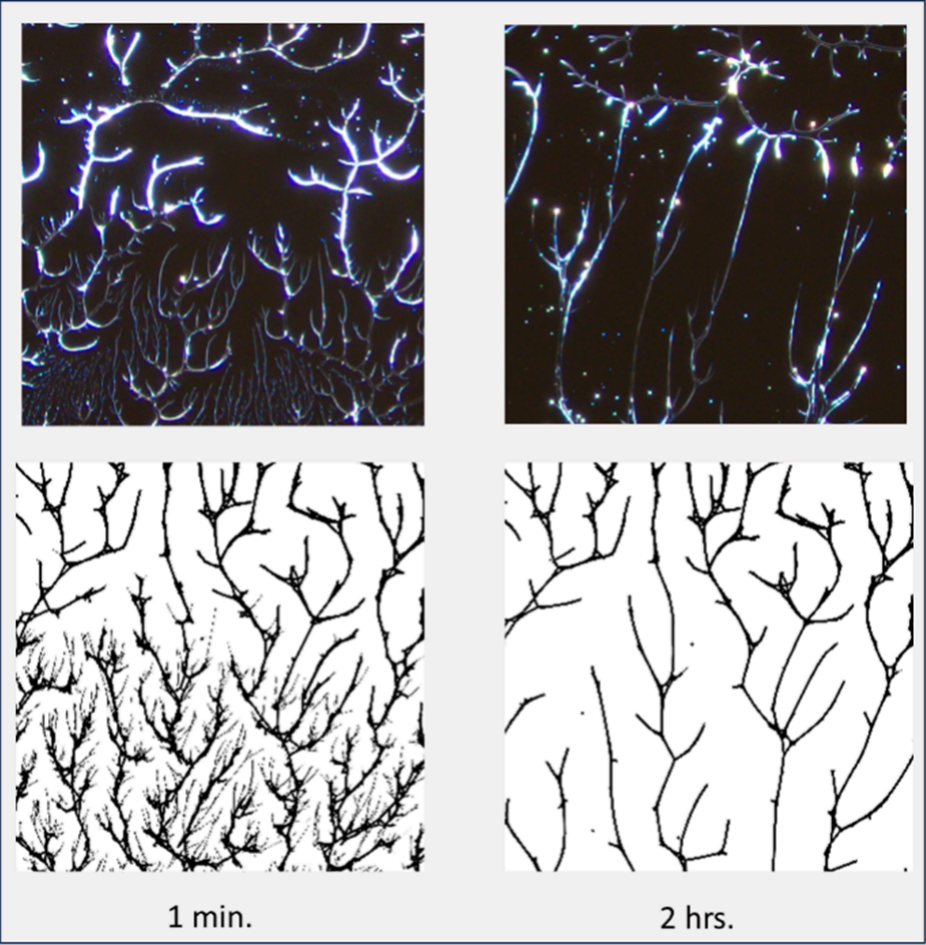
500x micrographs (top) of the extended drying 99%H samples at the edge of where the solvent was dispensed as well as corresponding simulation images (bottom) scaled appropriately.

We also find that our simulations capture certain finer-scale features. Figure 8 (top row) shows 500x micrographs of the development of 99%H samples at the edge of the dispensed solvent after 1 min and 2 h. In the corresponding simulations we see very similar patterns. After 1 min, we have a developing gap with the edge of the original polymer, and at the retreating edge of the gap we see very fine, secondary growth. As the boundary recedes further, any new growth contracts with it leaving only a skeletal structure connected to the original polymer structure.

**Figure 9:**
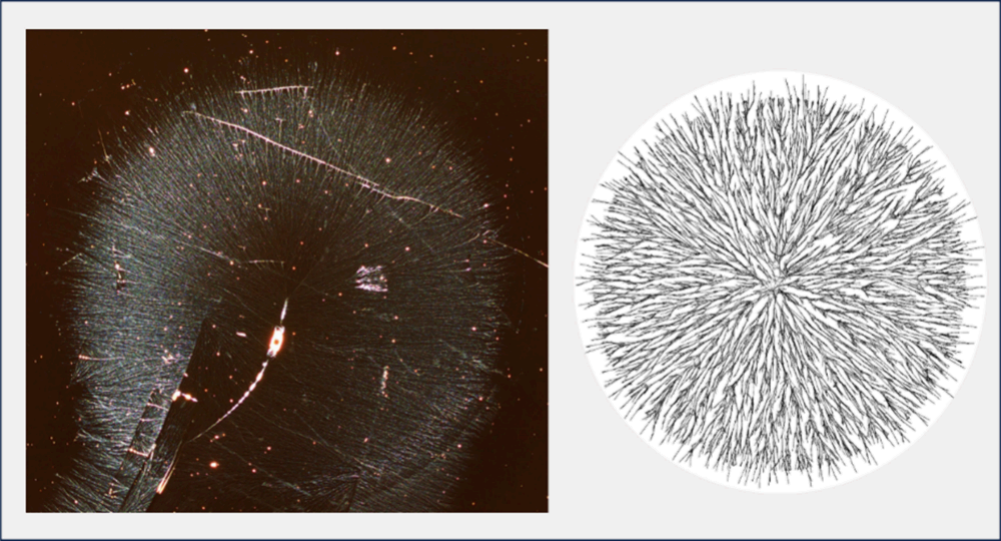
Five μL of PVOH^88%H^ adsorbed for 1 min prior to spin-coating (left image) and a similar structure (right image) seen in the extended drying simulation.

For the 88%H case, there are also finer scale features that our simulation captures. Figure 9 shows one sample of a thin film produced from adsorptive spin-coating of 5 μL of 88%H. As with the 99%H scenario, we hypothesize that the drying time was extended due to the smaller drop size, and we note that at the edge of film we see a needle-like structure with some needles extending beyond the more defined edge. This is also seen in the 88%H simulation.

Finally, two additional empirical contexts occurred through experimental irregularities, creating an opportunity to test whether or not the model can simulate contexts outside the typical process.

First, while multiple runs with current spin coaters show no difference in polymer morphology over a range of angular velocities between 900 and 6000 rpm (results not shown), earlier trials conducted with PVOH^99%H^ using an older spin coater (and reported by Qi et al.^3^) did show significant differences with changes in angular velocity. 200 μL of PVOH^99%H^ solution was dispensed onto a PDMS^49k^ substrate secured on the spin coater stage. After 1 min, the sample was spun at 900, 2200, 3500, 4800, or 6000 rpm for 1 min under nitrogen. It was noted that the older spin coater required a longer acceleration period, and as a consequence, a noticeable drop of solution remained in the center for an extended period. The radius of this center drop was inversely proportional to the spin rate.

In the area of the film where a drop of solution was resident for a longer period, we observe a network morphology that is less dense and coarser (Figure S4). It is hypothesized that this area experienced significantly longer drying time, something we can simulate by increasing the period of mobility. Further, the size of this region was inversely proportional to the spin rate.

To simulate the extended drying time in the center, the *mobility* was increased for particles within a certain radius of the center. *mobility* in the center was set at 400 and *mobility* in the outer annulus was set at 150. The radius of the boundary between the two areas can be adjusted to match the observed radius, but we did not attempt to model that aspect.

**Figure 10:**
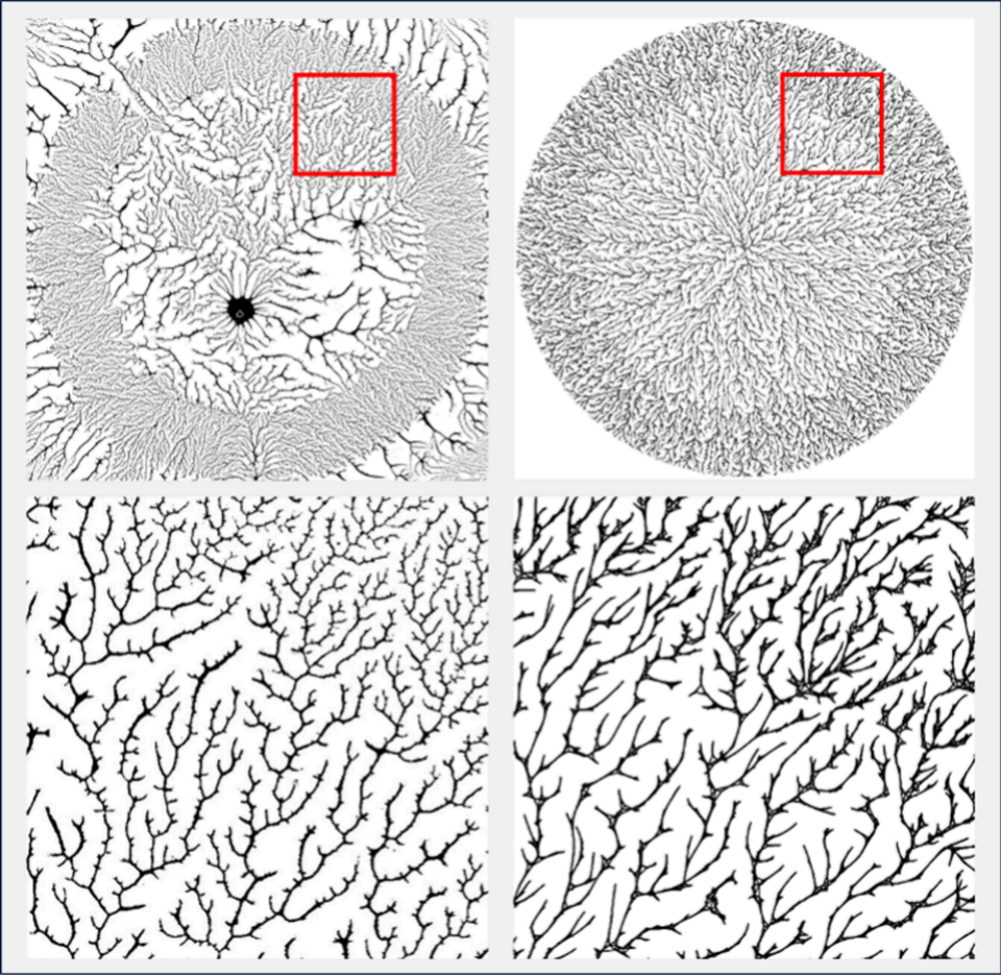
Experimental sample (left) and simulation results (right) for PVOH^99%H^ spin coated at 3500 rpm using an older spin coater as reported by Qi et al.^3^ To create the simulation on the right, two different values of mobility were used depending on distance from the center. The inner particles had a mobility of 400 and outer particles had a mobility of 150. The lower row shows a magnification of the inset and highlights the transition from a longer drying time to a shorter drying time. Images on the left are reproduced with permission from ref (3), copyright 2019 American Chemical Society.

Figure 10 shows the results of running a simulation where particles were assigned two different values for *mobility* based on their distance to the center (right side) and compares the resulting structure to one of the observed experimental trials (left side). A high value for *mobility* simulates a longer drying time, and increasing *mobility* in the center demonstrates a pattern similar to what we see in Figure S4. The lower row of Figure 10 shows a magnified inset on each of the images, demonstrating similar network characteristics in the transition zone.

Finally, our simulation efforts have largely focused on spin-coated polymers that demonstrate a radial growth pattern. However, when smaller drops have been used that do not cover the entire PDMS substrate, interesting fractal growth patterns have been observed along the liquid exit path, a region of polymer aggregation where the flow of the liquid follows a (largely) linear path. The previously described experiment with variable volumes of PVOH solution produced fractal structures outside of the footprint of the dispensed solution. These structures were not the target of the original experiment and occurred along the exit path of the solution in the initial stages of spin coating. The experimental setup is identical to that described above, but the area of analysis is now outside the footprint of the dispensed solution, an area that only came into contact with PVOH as the solution was spun off in a clear, spiraling path.

In this region, polymer growth produces “fishbone” like structures with side branching perpendicular to the flow (Figure S5). Our working hypothesis to explain this phenomenon is that the availability of particles to bond is dependent on the moisture level. Bond formation does not begin to happen until most of the moisture is removed, and so particles in the flow path must wait until the front edge of the flow moves past before they are available to bond.

To model this, we assume that nucleation sites form as the solvent flows along the path and then become amenable to bond as the liquid is removed along a triangular front (Figure S6). This means that particles only become available for bonding after the moving front has passed them. Model parameters were added to control the angle of the front and its speed. The goal of this simulation was to demonstrate the ability of the model to replicate the observed patterns under atypical conditions by making reasonable changes to the model.

**Figure 11:**
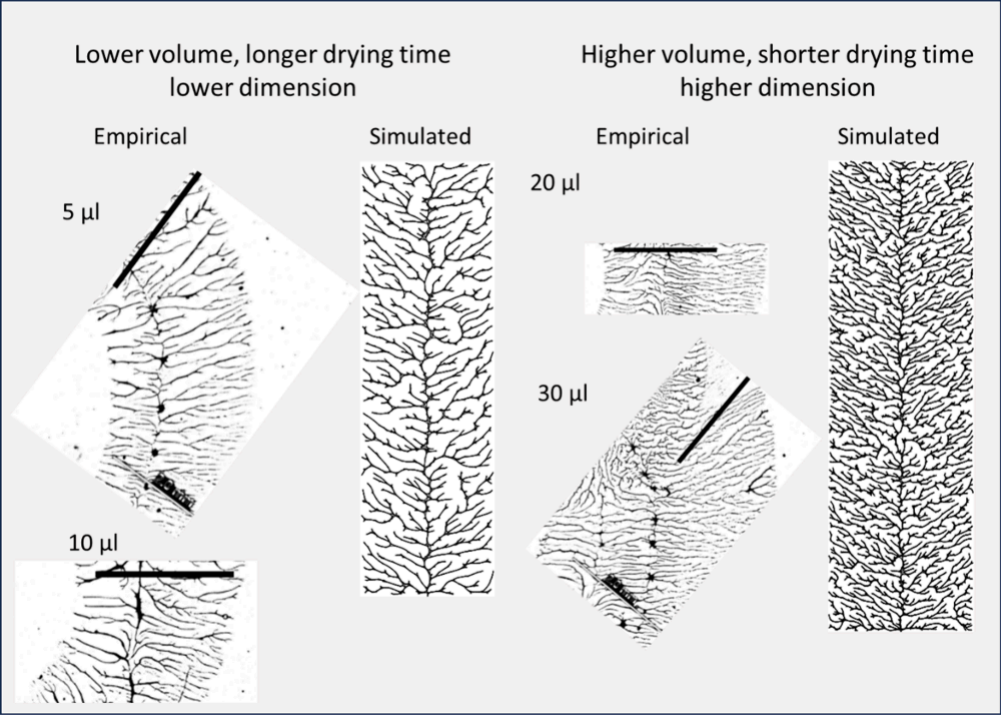
Observed and simulated “fishbone” structures. The empirical images show the exit line from spin coating a PVOH^99%H^ solution. Images on the left are from smaller initial volumes of solution while those on the right started with larger volumes. Simulated images were produced by assuming that particles became available for bonding as the flow of liquid recedes. The image on the left was made with 10,000 particles and *mobility* = 600 representing a long drying time. The image on the right was made with 25,000 particles and *mobility* = 400, a shorter drying time but still longer than the base case (*mobility* = 200).

Figure 11 shows the simulation of such a fishbone structure under two different conditions—a smaller quantity of particles with a longer drying time and a higher quantity of particles with a shorter drying time, though in both cases the drying time is thought to be considerably longer than the base case with PVOH^99%H^ spun at 6000 rpm. In this case, it was not the goal to provide an exact simulation of the structure but rather to show that a few reasonable adjustments to the model could result in patterns similar to what was observed, patterns that are also noticeably different from most other experimental output.

This work offers a new mathematical framework for modeling fractal growth processes. Previously, several different models of fractal growth have been utilized to simulate aggregation and crystallization as well as other methods of fractal formation in materials science. Most notably, the DLA model has been widely used and adapted.^22^ Amir et al.^13^ use the DLA model as well as an L-system dialectic to simulate observed fractal structures in polymer films and demonstrate a reasonable fit with the development of the fractal dimension over time. With modifications (and concomitant increases in complexity), the DLA model is able to simulate differences in many experimental contexts. Li et al.^23^ use a modified 2D DLA model to simulate a variety of fractal formations observed in chemical vapor deposition, and by constraining the domain of the random walkers in the DLA model to simulate variation in viscosity, Zhang and Liu^24^ were able to simulate significant variability in viscous fingering.

In this work, we have applied a relatively simple model of fractal growth to the simulation of spin-coated polymers. We started by showing the ability of the model to simulate a base case of spin-coated PVOH^99%H^, but this in and of itself is only suggestive of the ability of the model to simulate the broader experimental system. An effective model of an experimental system should have clearly interpretable processes and parameters that are capable of simulating the behavior of the system under a variety of empirical conditions. Further, the changes made to model parameters or processes to simulate different experimental outputs should be consistent with the underlying differences in the experimental systems.

The triangular growth model used in this system has a relatively small number of parameters and mechanisms that all have a clear interpretation in terms of polymer aggregation and bonding. The model components can be rigorously mapped to the core elements of the experimental system. Model particles represent polymer nuclei at a level of aggregation just prior to bonding. The triangular “bonds” represent the bonds formed between nuclei during bonding. Prebonding nuclei may vary in density and size, and thus the model allows for variation in particle density with an inversely related variation in the bond width.

The formation of new bonds is not instantaneous but rather represents a growth process. Thus, in the model, bonds are formed at a specified bonding rate and changes in bonding rate affect the fractal morphology with lower bonding rates leading to lower-dimensional, less-dense growth.^14^ A similar effect is documented by Wang et al.^7^ in which increasing the number of POSS heads on an amphiphile chain increases the restrictions on crystallization. As a consequence, they observe lower-dimensional fractal growth (Figure 10 in Wang et al.^7^).

**Figure 12:**
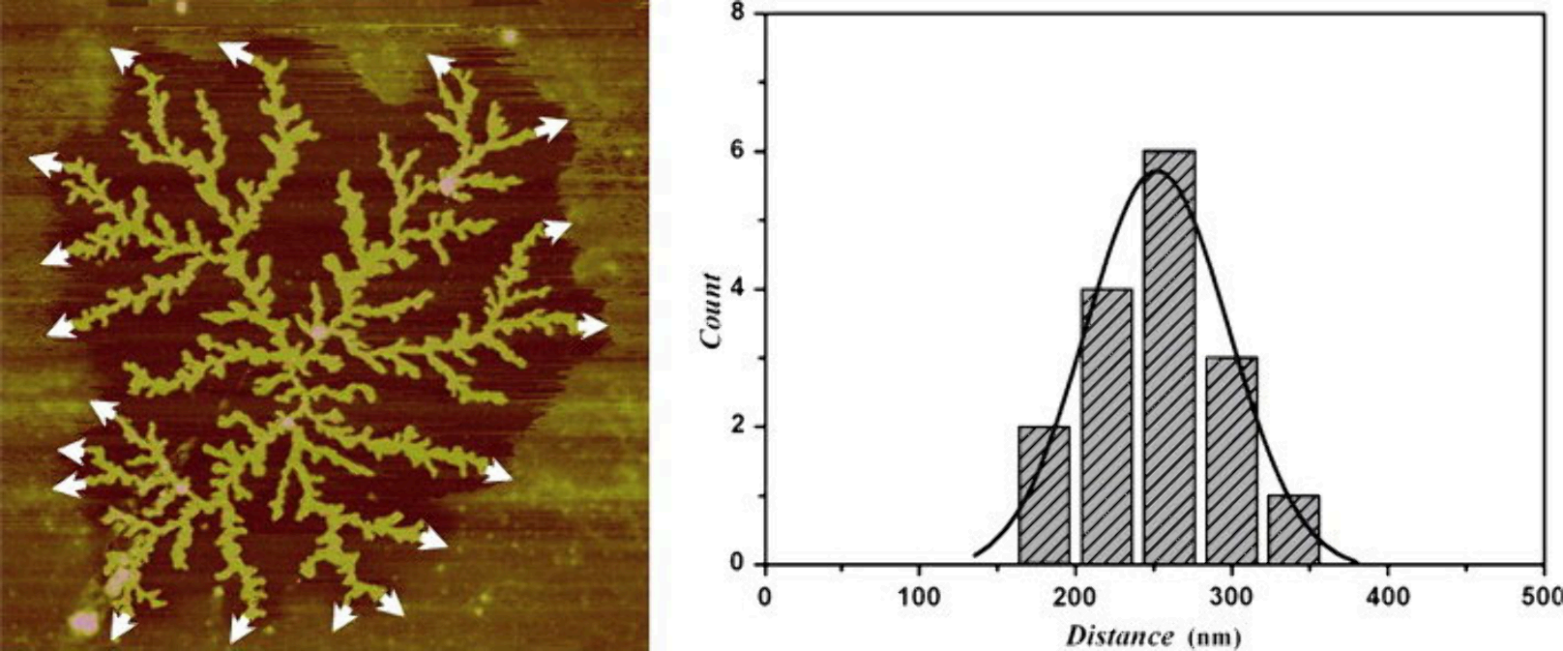
Fractal development of a poly(ethylene oxide) monolayer crystal showing a clear gap between the surrounding amorphous layer and the developing crystal. Figure reproduced with permission from ref (23). Copyright 1998, American Chemical Society.

Similarly, there is a spatial limit to the range at which bonds can be formed, represented in the model by the bonding range. And as the polymer bonds, the network mass also tightens and contracts, at least until such time as the polymer fully dries. This period of contraction prior to fully drying is represented by mobility in the model. These two phenomena are well documented by Ma et al.^25^ in the fractal growth observed in a poly(ethylene oxide) monolayer. They document the time-dependent growth of the polymer fractal and show a clear boundary between the aggregate and the surrounding amorphous layer as well as a measurable gap between the two (Figure 12). This picture of fractal development is much more consistent with our triangular growth model—in which the growing mass bonds to surrounding particles and pulls them toward the mass—than it is to the DLA model in which such a gap should not be present.

The differences between the various experimental contexts explored in this work can all be interpreted as differences in the system factors detailed above and thus can be simulated by making appropriate changes to their corresponding model components. We have shown that these changes—when effected in the model—lead to simulation results that closely match the observed differences in the polymer fractal morphology. In particular:

- By increasing the density of nuclei, we were able to simulate the finer, denser growth observed in spin coating with a PVOH^88%H^ solution.
- By changing the number of particles and modifying the mobility in line with hypothesized differences in drying times, we were able to model the changes observed when using different volumes of PVOH^99%H^ including accurately capturing the gradient in fractal dimension.
- By dissolving a portion of the bonds and allowing the fractal to regrow with an increased density of nuclei, we were able to closely simulate the effects of solvent annealing for 88%H and 99%H.
- By allowing an annealed structure to have mobile nuclei at the edge of a receding drying front, we were able to model multiple characteristics observed in the extended drying experiments including capturing differences observed at different time scales.
- When an equipment anomaly led to an extended drying period in the middle of the structure, we were able to accurately model the differences by increasing *mobility* within the observed radius. Effecting an accurate simulation by changing only this one parameter provides evidence that it is indeed a change in drying time (and the related contraction) that drives the observed empirical differences.
- And when the flow path of the solution during spin coating produced a novel fractal structure, we were able to simulate the development of this structure by modifying which nuclei are available for bonding in a manner consistent with our understanding of the retreating flow of solution.

None of the polymer fractal simulation models discussed earlier has been shown to be capable of matching the observed variability in fractal formation that results from experimental modifications. Indeed, we are not aware that any such modeling has happened before. It is the case that the DLA model has been widely adapted to different contexts, and this often involves variations to the model.^26−29^ One of the better-studied is the dielectric breakdown model which places the DLA framework within an electric field.^30^ Generally, such modifications result in significant changes to model dynamics and also a significant increase in complexity. They are useful for modeling different systems, but they do not equate to using the same model framework to produce a diversity of behaviors.

By and large, the different systems modeled in this work are simulated only by making changes in model parameters or, in the case of annealing and the extended drying experiments, allowing the model to work twice to mimic the two-step empirical processes. No additions are made to the model that significantly change its structure. The closest we come to a dramatic change in the model is in changing the pattern of available particles in simulating the spin-coating exit flow, and we view this as an edge case.

## Conclusions

A model such as ours has the ability to provide evidence for or against different hypotheses, especially regarding aggregation phenomena that are not directly observable. The differences in structure observed between PVOH^99%H^ and PVOH^88%H^ can be explained by two different changes in the model—increasing the bonding rate or increasing the density of nucleation sites. While both hypotheses have theoretical justification, the similar changes in formation that are observed when the polymer is annealed cannot be successfully modeled by changes in bonding rate. They can, however, be simulated by making changes in density. While this is far from conclusive, it suggests that nucleation density, or the extent of polymer preaggregation, may function as a critical driver of thin film morphology.

There is also room for further variability in behavior and processes which will allow us to simulate additional experiments and test future hypotheses. As an example, work is currently underway to investigate the fractal structure produced when the PVOH solution is allowed to air-dry rather than be spun off. Initial results (not shown) suggest that empirical results are in line with the predictions of the model. In future work, we will compare similar *a priori* predictions made by the model to empirical observations.

Simplicity should be, and is, one of the most highly desired traits in models. While it is often a necessity to add greater detail to a model based on the modeling objectives, such increased complexity comes at a cost.^31,32^ In this work we have utilized a conceptually simple dynamic model to simulate experimental variability in an important empirical setting where many questions exist regarding the underlying dynamics. This provides some evidence for hypotheses regarding the processes involved and also gives us the ability to make predictions about additional experiments. Perhaps most importantly, it clearly demonstrates a simple potential mechanism underlying the range of complex behaviors and fractal morphologies observed in this system. If further work supports this mechanism, it can lead to diagnostic and design algorithms for these polymer thin films. Future work will focus on developing effective quantitative statistics for validating and parametrizing models.

## Supporting Information Available

Representative images of model development. Sample images from model behavior space. Images showing the effects of annealing on polymer fractal morphology. Sample polymers produced by varying spin rate. Representation of the growing front for simulating PVOH exit path. Sample images showing the PVOH exit path in spin coating (PDF)
