# Supplementary material for: Simulation of Polymer Fractal Formation Using a Triangular Network Growth Model
Source: Langmuir. 2024 Sep 27;40(40):21253–62. doi: 10.1021/acs.langmuir.4c02939 (PMC11465768; doi:10.1021/acs.langmuir.4c02939)
Supplement: Supplementary file 1 — la4c02939_si_001.pdf [file la4c02939_si_001.pdf]

Supporting Information

Simulation of polymer fractal formation using a triangular network growth model

Kenneth Mulder[1,3*], Hannah Heierhoff[1], Sophia M. Lee[2], Jeannie Ji-Ying Tsou[2], Wei Chen[2]

[1]Department of Mathematics and Statistics, Mount Holyoke College, South Hadley, Massachusetts, USA 01075

[2]Department of Chemistry, Mount Holyoke College, South Hadley, Massachusetts, USA 01075

[3]School of Natural Sciences, Hampshire College, Amherst, Massachusetts, USA 01075

* Corresponding author

Email: kmulder@hampshire.edu

Contents

Representative images of model development.

Sample images from model behavior space.

Images showing the effects of annealing on polymer fractal morphology.

Sample polymers produced by varying spin rate.

Representation of the growing front for simulating PVOH exit path.

Sample images showing the PVOH exit path in spin coating.

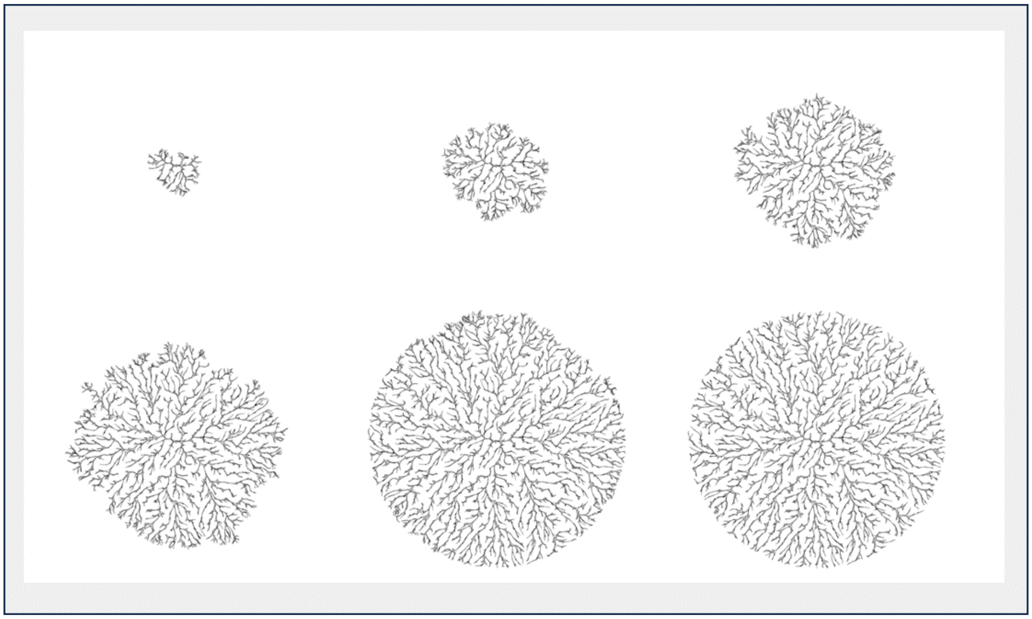

Figure S1 – An example of model growth. Parameter settings are *mobility* = 200, *bond-range* = 2.2, *bond-rate* = 0.03, *density* = 1.0 and *n-particles* = 10^5. Images are shown at 300, 600, 900, 1200, 1500, and 1891 timesteps.

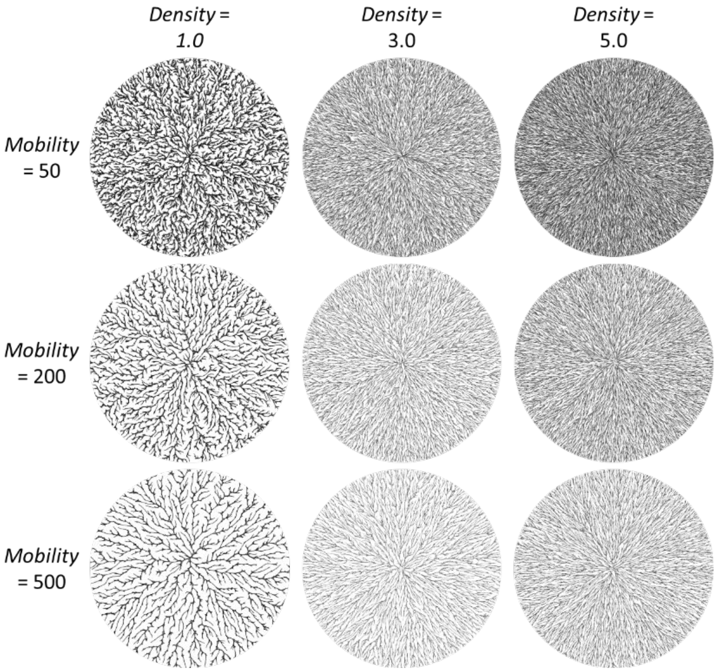

Figure S2 – Changes in model behavior with respect to changes in mobility and density. Increasing density leads to more outward, radial growth and more uniform coverage of the disk. At low density values, branches have more curvature. Increasing mobility reduces the density of the final structure and leads to shorter side branches. The values for the other parameters are *bond-range* = 2.2, *bond-rate* = 0.03, and *n-particles* = 10^5.

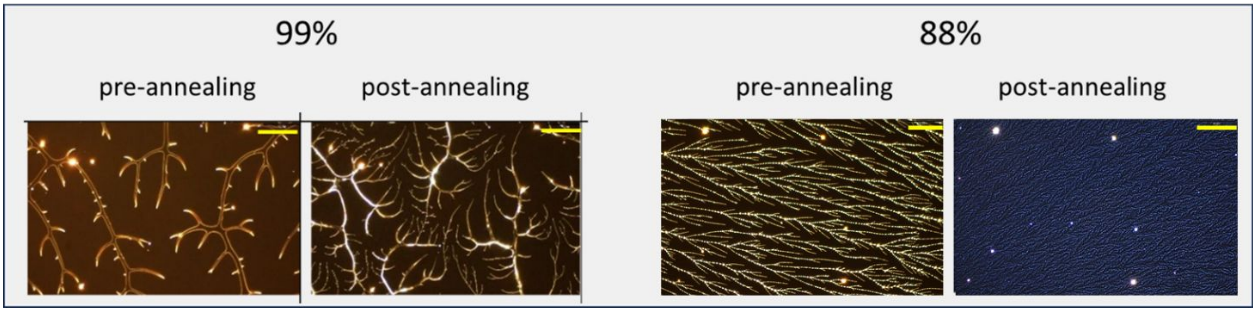

Figure S3 – Spin-coated polymers made from PVOH solution with 99%H (pair on left) or 88%H (pair on right). Within each pair, the image on the left shows the polymer prior to solvent annealing and the image on the right shows the same polymer after 1 minute of solvent annealing and 1 minute of spin coating. Annealing and reducing the degree of hydrolysis of PVOH (%H) show similar effects in terms of finer, denser fractal growth. The yellow scale bars have lengths of 20 μm.

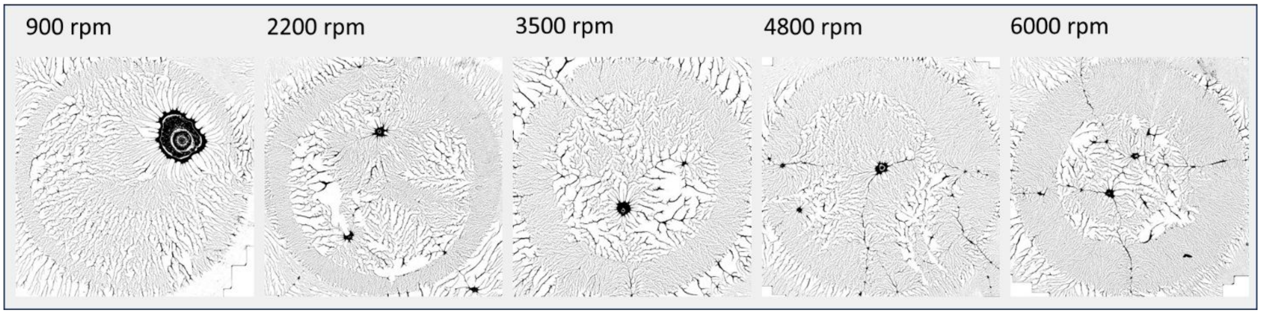

Figure S4 – Results from Qi et al. (2019) of thin film polymers produced using an older spin coater set to different spin rates. Micrographs of the entire polymers were stitched together from sectional optical images (50x) using Autopano Giga. The older machine has a noticeable time lag before reaching the desired angular velocity, and during this time, it was noted that a drop of solution was resident for a period of time in the center of the resulting film. Image reproduced with permission from reference 3, copyright 2019 American Chemical Society.

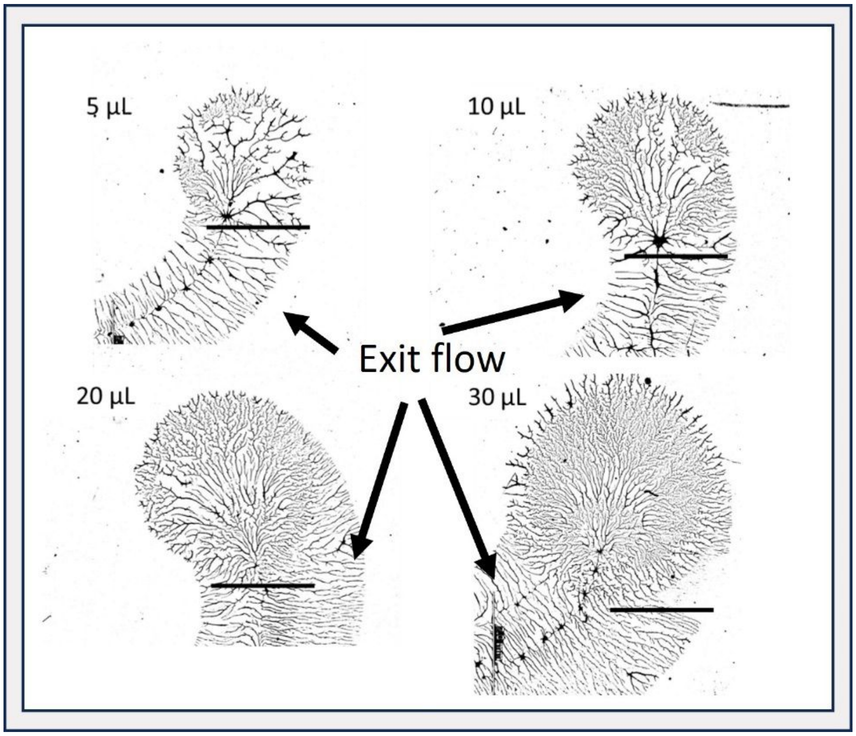

Figure S5 – Spin-coated PVOH$^{99\%H}$ polymer fractals created from different starting volumes of solution. The volume of solution applied is shown to the upper left of each image. The initial adsorption area did not cover the entire film, and solvent that was spun off left a "fishbone"-shaped fractal structure along the exit path. As with the center region, we hypothesize that the drying time along the exit path was longer for smaller quantities of solution.

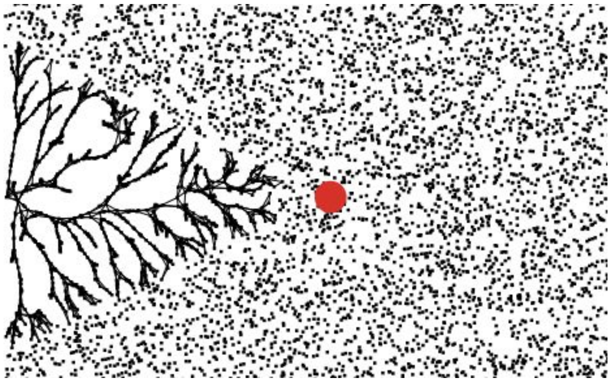

Figure S6 – Representation of the growing front of the polymer fractal as the solution is spun off in a stream. The red dot shows the leading point of the triangular growth region. Particles outside the region are not yet available for bonding.
